# The circadian regulator PER1 inhibits osteoclastogenesis by activating inflammatory genes

**DOI:** 10.1101/2025.09.18.677145

**Published:** 2025-09-19

**Authors:** Nobuko Katoku-Kikyo, Elizabeth K. Vu, Samuel Mitchell, Ismael Y. Karkache, Elizabeth W. Bradley, Nobuaki Kikyo

**Affiliations:** 1 Stem Cell Institute, University of Minnesota; 2 Department of Genetics, Cell Biology, and Development, University of Minnesota; 3 Department of Orthopedic Surgery, University of Minnesota

**Keywords:** Bone mass, circadian rhythms, inflammasome, inflammation, osteoclast, osteoimmunology, Per1

## Abstract

Disruption of circadian rhythms predisposes shift workers to many chronic conditions, including osteoporosis. However, the effects of disrupted circadian rhythms on bone remodeling remain largely unknown. Here, we show that one of the core circadian regulators PER1 inhibits osteoclastogenesis by upregulating genes involved in inflammation. The conditional knockout of *Per1* in osteoclasts and related cells resulted in decreased bone mass in the femurs of mice, along with increased osteoclasts and decreased osteoblasts. Osteoclastogenesis was also promoted by *Per1* depletion in vitro with 17 downregulated inflammatory genes. Eight of these genes were known to promote or inhibit osteoclastogenesis depending on the stage of osteoclastogenesis and the presence or absence of infection. The knockdown of three of these genes, which were involved in the inflammasome pathway, promoted osteoclastogenesis, mirroring the effects of *Per1* knockout and offering a mechanistic explanation for the *Per1*-mediated inhibition of osteoclastogenesis. These results were not observed following the depletion of a paralog *Per2*. *Per1* knockout mice maintain general circadian rhythms, unlike arrhythmic *Per1*/*Per2* double knockout mice. This gives credence to *Per1* as a selective target for therapeutic interventions without disrupting the circadian rhythms. This study uncovered a link between a circadian regulator and osteoclastogenesis in the broader context of osteoimmunology. Our findings may be mechanistically relevant to inflammatory bone diseases influenced by circadian rhythms, such as rheumatoid arthritis and osteoarthritis, as well as other bone diseases predisposed by chronic circadian disruption.

## Lay summary

Disruption of circadian rhythms is a risk factor for many chronic diseases, including osteoporosis, among shift workers; however, the underlying mechanisms remain largely unknown. In this study, the depletion of *Per1*, a core circadian regulator, resulted in an increase in bone-resorbing osteoclasts and a decrease in bone mass in mice. These changes were accompanied by a decrease in the expression of inflammatory genes that promoted the formation of osteoclasts upon depletion. This study revealed a link between circadian rhythms and bone loss, with inflammatory genes serving as mediators, which could provide a basis for future therapeutic interventions.

## Introduction

Disruption of circadian rhythms due to frequent travel, shift work, or excessive exposure to artificial light increases the risk of osteoporosis,^1,2^ a condition characterized by an imbalance in bone formation by osteoblasts and bone resorption by osteoclasts, both of which are under circadian control^3^. Mammalian circadian rhythms are regulated by the central clock located in the suprachiasmatic nucleus of the hypothalamus and peripheral clocks, which are ubiquitously expressed throughout the body.^4,5^ The central clock is entrained by light signals transmitted from the retina and synchronizes peripheral clocks via the autonomic nervous system and various hormones. Peripheral clocks are also entrained by other stimuli, such as food intake and sleep-awake signals. All these clocks are maintained by two feedback loops centered on the CLOCK/BMAL1 transcription factor complex. This heterodimer binds to the E-box in hundreds of target genes and activates their transcription. The target genes include *Cry* (*Cry1* and *Cry2*) and *Per* (*Per1*, *Per2*, and *Per3*), which form the CRY-PER heterodimer and inhibit CLOCK/BMAL1 through direct binding. Subsequently, both CRY and PER are degraded via the proteasomal pathway, enabling CLOCK/BMAL1 to resume transcription of the target genes and completing the first feedback loop with a 24-hr period. In the second feedback loop, CLOCK/BMAL1 activates the transcription of the *Ror* (retinoic acid receptor-related orphan receptor) and *Nr1d* (Rev-erbs or reverse orientation c-erbA) genes, whose proteins then bind to the *Bmal1* promoter and activate (ROR) or inhibit (NR1D) transcription.

Circadian regulation of bone remodeling has been primarily demonstrated through three lines of evidence.^3,6^ First, serum and urine levels of bone turnover markers representing bone resorption (such as crosslinked C-terminal telopeptide of type I collagen [CTX]) and bone formation (such as procollagen type 1 N-terminal propeptide [P1NP]) exhibit circadian rhythms. Second, RNA-seq and quantitative reverse transcription and PCR (qRT-PCR) demonstrate circadian expression of bone marker genes, such as *Runx2*, *Nfatc1*, *Ctsk*, *Tnfrsf11a* (*Rank)*, and *Tnfrsf11b* (*Opg*) in the mouse calvaria, tibia, and femur. Third, bone cell-specific knockout (KO) of circadian regulators disrupts bone mass. For example, osteoblast-specific KO of *Bmal1* decreases bone mass by modulating the RANKL signaling pathway.^7^ In contrast, osteoclast-specific KO of *Bmal1* increases bone mass,^8^ although another study was unable to replicate this finding.^9^ However, since the depletion of *Bmal1* completely abolishes circadian rhythms, these phenotypes could include general consequences of the lack of circadian rhythms, in addition to the intended *Bmal1*-specific effects as mentioned earlier.^10^

We depleted *Per1* or *Per2* in the osteoclasts of mice in the current study for two reasons. First, singular germline KO mice for either gene can maintain circadian rhythms with a period of approximately 22 hr, unlike arrhythmic double KO mice,^11^ addressing the problem mentioned above. Although germline KO of *Per1* or *Per2* does not result in an overt skeletal phenotype,^13^ this does not exclude hidden roles of the genes in osteoclastogenesis. The roles of non-*Bmal1* circadian regulators, including *Per*, in osteoclastogenesis remain poorly understood. Second, *Per1* KO promotes the production of pro-inflammatory cytokines in macrophages under inflammatory conditions as we reported before^12^, suggesting that *Per1* KO might affect osteoclasts as well because of the shared linage between macrophages and osteoclasts.

In this study, we used *Cx3cr1* promoter-driven *Cre* mice to conditionally KO (cKO) *Per1* or *Per2* in the monocyte/macrophage lineages. We first compared the bone phenotypes of *Per1* cKO and *Per2* cKO mice using micro-computed tomography (micro-CT) and histology. We then used in vitro osteoclastogenesis to verify the cell-autonomous effects of *Per1* cKO. Subsequently, transcriptomics, gene knockdown (KD), and circadian synchronization led us to identify a group of genes that could explain the phenotypes of *Per1* cKO. Collectively, these results revealed osteoimmunology as a major link between *Per1* and osteoclasts.

## Materials and Methods

### Generation of *Per1* and *Per2* cKO mice

*Per1* cKO mice were created by crossing the *Cx3cr1* promoter-driven *Cre* mice (B6J.B6N(Cg)-*Cx3cr1*^*tm1.1(cre)Jung*^/J, Jackson Lab, 025524) with the inducible *Per1* KO mice *Per1*^*fl/fl*^.^14^ The resulting *Cx3cr1-Cre*^*tg/wt*^*;Per1*^*fl/fl*^ and *Cx3cr1-Cre*^*wt/wt*^*;Per1*^*fl/fl*^ mice were used as *Per1* cKO and littermate control mice (*Per1* Cont), respectively. Likewise, *Cx3cr1*-*Cre* mice and *Per2*^*fl/fl*^ mice (Medical Research Council, UK, Per2^tm1c(EUCOMM)Hmgu^) were crossed to establish *Cx3cr1-Cre*^*tg/wt*^*;Per2*^*fl/fl*^ and *Cx3cr1-Cre*^*wt/wt*^*;Per2*^*fl/fl*^ mice as *Per2* cKO and littermate control mice (*Per2* Cont), respectively. Genotyping primers and the sizes of the PCR products are listed in Supplementary Table 1 based on the protocols provided by the sources of the mice. The mice were housed under a 12 hr light-12 hr dark cycle in an accredited facility with water and food provided *ad libitum*. All mouse protocols were approved by the Institutional Animal Care and Usage Committee of the University of Minnesota (2410–42491A). All mouse experiments comply with the standards set by the Guide for the Care and Use of Laboratory Animals published by the US National Institute of Health.

### Micro-CT scan analysis of the bones

Micro-CT scanning of the femur was performed as previously described.^15^ Specifically, the femurs prepared from 12-week-old mice were fixed in 10% formalin in phosphate-buffered saline (PBS) for 24 hr and stored in 70% ethanol at 4°C. Mico-CT was performed with an XT H 225 CT scanning system (Nikon Metrology). The scan settings were as follows: 120 kV, 61 μA, 720 projections at two frames per projection with an integration time of 708 ms, an isometric voxel size of 7.11 μm with a 1 mm aluminum filter, and 17 min per scan. Bone morphometric analysis was done with SkyScan CT Analyzer (CTAn, Brucker micro-CT) and the results were described with standard symbols.^16^ Each scan volume was reconstructed with CT Pro 3D program (Nikon Metrology), converted to bitmap data with VGStudio MAX 3.2 (Volume Graphics), and reoriented with DataViewer (SkyScan, Buker micro-CT) for qualitative analysis. 3D analysis of trabecular bones was done at the distal metaphysis 0.7 mm proximal to the growth plate, extending 1.5 mm toward the bone diaphysis. 3D analysis of the cortical bones was done in a 0.5 mm section in the mid-diaphysis 4 mm from the growth plate.

### Histomorphometry of bone sections

The tibiae prepared as described above were decalcified in 15% EDTA for 14 days, embedded in paraffin, and sectioned at 7 μm in thickness. The TRAP staining kit (Sigma Aldrich, 387A-1KT) and Masson’s trichrome staining kit (Polyscience, 25088) were used to detect osteoclasts and osteoblasts, respectively. The numbers of osteoclasts and osteoblasts were counted and the ratios between these cells and bone perimeter were calculated with ImageJ.

### Osteoclast differentiation in vitro

Osteoclasts were prepared from bone marrow monocytes and macrophages (BMMs) as previously described with some modifications.^17^ On day 1, bone marrow cells were flushed from the femur and tibia of 8- to 12-week-old mice with PBS and centrifuged at 190 x *g* for 5 min. The precipitated cells were resuspended with 1X Red Blood Cell Lysis Buffer (eBioscience, 00-4333-57) and incubated at 25°C for 5 min. After centrifugation under the same conditions, the precipitated cells were resuspended with Monocyte Medium (25 ng/ml M-CSF [Shenandoah, 100–03], 10% heat-treated fetal bovine serum [FBS], and phenol red-free αMEM [Thermo Fisher, 41061–029]). Cells were incubated with 5% CO_2_ at 37°C overnight. On day 2, non-adherent cells were harvested and cell clusters were removed with a 70 μm cell strainer (Falcon, 352350). The cells were centrifuged, resuspended in Osteoclast Medium (Monocyte Medium with 100 ng/ml RANKL [Cell Signaling Technology, 68495]), and seeded at 8×10^5^ cells/400 μl/well in a 48-well plate. On day 4, 200 μl of the medium was added. On day 5, the medium was replaced with 400 μl of fresh medium. On day 6, the cells were fixed with 4% paraformaldehyde in PBS for TRAP staining. The total number of osteoclasts, defined as TRAP-positive cells with more than two nuclei, in a well were manually counted. The sizes of osteoclasts were measured with ImageJ.

### Enzyme-linked immunosorbent assay (ELISA) of IL-1β and lipopolysaccharide (LPS)

Culture supernatant of osteoclasts on day 6 were applied to ELISA of IL-1β (Lumit IL-1β immunoassay, Promega, W7010) and LPS (ToxiSensor chromogenic LAL endotoxin assay kit, GenScript, L00350Y) following each instruction.

### Synchronization of circadian rhythms

On day 5 of the differentiation of osteoclasts, the cells were treated with Synchronization Medium (50% heat-inactivated horse serum in Osteoclast Medium instead of FBS) for 1 hr at 37°C with 5% CO_2_, washed with PBS at 37°C twice, and cultured with fresh Osteoclast Medium.^18^ The time of the completion of these procedures was defined as 0 hr post-synchronization. The cells were cultured at 37°C with 5% CO_2_ and harvested for qRT-PCR every 4 hr starting from 24 hr post-synchronization to wait for the recovery from the synchronization as previously recommended.^19^ Circadian rhythmicity of the qRT-PCR values was evaluated with Cosinor.Online (https://cosinor.online/app/cosinor.php).^20^

### Bone resorption assay

The bone resorption assay followed a published protocol with some modifications.^17^ On day 2 of the osteoclast differentiation, the cells were seeded on top of a bone slice (Immunodiagnostic Systems, DT-1BON1000–96) at 3.2×10^5^ cells/well in a 48-well plate in Osteoclast Medium to induce osteoclastogenesis as described above. The medium was replaced every 3 days after day 6. On day 14, cells were lysed with 10% bleach and the bone slices were washed with deionized water three times. The bone slices were incubated in 200 μl deionized water for 1 hr at 25°C twice and stained with Toluidine blue for 30 sec. The bone slices were washed with deionized water three times and dried. The resorption area was analyzed with ImageJ.

### qRT-PCR

qRT-PCR was performed as previously described.^12^ RNA was extracted from cells using a Quick RNA Microprep kit (Zymo Research, R1051) and purity was assessed using a microvolume spectrophotometer DS-11 FX+ (Denovix). cDNA was synthesized with ProtoScript II Reverse Transcriptase (New England Biolabs, M0368L). qPCR was performed with the primers listed in Supplementary Table 2 and qPCRBIO SyGreen Blue Mix Lo-ROX (Genesee Scientific, 17–505B) on a Mastercycler realplex^2^ thermocycler (Eppendorf). PCR conditions were as follows: initial denaturation at 95°C for 2 min, 40 cycles of 95°C for 5 sec - 60°C for 30 sec - 72°C for 30 sec, and a melting curve step to check the specificity of the amplification. mRNA expression levels were analyzed by normalizing expression values to glyceraldehyde 3-phosphate dehydrogenase (*Gapdh*) expression. Mean ± SEM of biological triplicates with technical triplicates each was calculated.

### Gene KD with siRNA

BMMs were transfected with 10 nM siRNA (Supplementary Table 3) with 1 ul DharmaFECT 1 Transfection Reagent (Dharmacon, T-200-01) for 5 hr on day 4 and 5 during osteoclast differentiation described above. The cells were harvested on day 6 for qRT-PCR and TRAP staining.

### Transfection of circadian genes into RAW264.7 cells

RAW264.7 cells (ATCC, TIB-71) were transfected with empty vector or plasmids encoding *Clock*, *Bmal1*, *Per1*, and *Per2* as follows.^21^ Cells were seeded at 8×10^4^ cells/well in a 48-well plate on day 1 in 10% FBS in DMEM. On day 2, 1.6 μg of the plasmid was transfected with 2 μl Lipofectamine LTX and 1.5 μl PLUS Reagent (Invitrogen, 15338030) following the instructions. The cells were incubated at 37°C with 5% CO_2_ and harvested 48 hr later for qRT-PCR.

### RNA-seq

RNA-seq and data analysis were also performed as previously described.^22^ Total RNA was prepared from day 6 osteoclasts and concentration and RNA integrity number were quantified with an Agilent BioAnalyzer 2100. mRNA was purified with poly-T oligo-attached magnetic beads and cDNA was synthesized using random hexamer primers. Non-directional libraries were prepared and completed by end repair, A-tailing, adapter ligation, size selection, amplification, and purification. The quality and quantity of the libraries were checked via real-time PCR and a Bioanalyzer. The libraries were sequenced on an Illumina platform and paired-end reads were generated.

Raw reads of the fastq format were processed through in-house perl scripts. Paired-end clean reads were aligned to the reference genome Mus musculus GRCm38 (ftp://ftp.ensembl.org/pub/release-94/fasta/mus_musculus/dna/Mus_musculus.GRCm38.dna.primary_assembly.fa.gz and ftp://ftp.ensembl.org/pub/release-94/gtf/mus_musculus/Mus_musculus.GRCm38.94.gtf.gz) using Hisat2 v2.0.5 (https://daehwankimlab.github.io/hisat2/). featureCounts v1.5.0-p3 (http://subread.sourceforge.net/) was used to count read numbers mapped to each gene. Differential expression analysis was performed using the DESeq2 R package (1.20.0) (https://www.r-project.org/). Genes with log2 fold change > 0.58 of < –0.58 (> 1.5-fold) and an adjusted p-value < 0.05 were assigned as differentially expressed. Enrichment of specific gene pathways in differentially expressed genes were identified by applying the clusterProfiler R package (https://www.r-project.org/) to the databases of Gene Ontology (http://www.geneontology.org). Adjusted p-value < 0.05 was considered significantly enriched.

### Statistical Analysis

Unpaired two-tailed t-tests and two-way ANOVA with Tukey’s method of multiple comparisons were used as stated in the figure legends. Mean ± SEM obtained from biological replicates of the numbers are indicated in each figure. Box plots show median and interquartile range (25th – 75th percentile). GraphPad Prism 10 (GraphPad Software) was used in statistical analysis.

## Results

### *Per1* cKO, but not *Per2* cKO, decreased bone mass and increased osteoclasts in male mice

**Figure 1.**
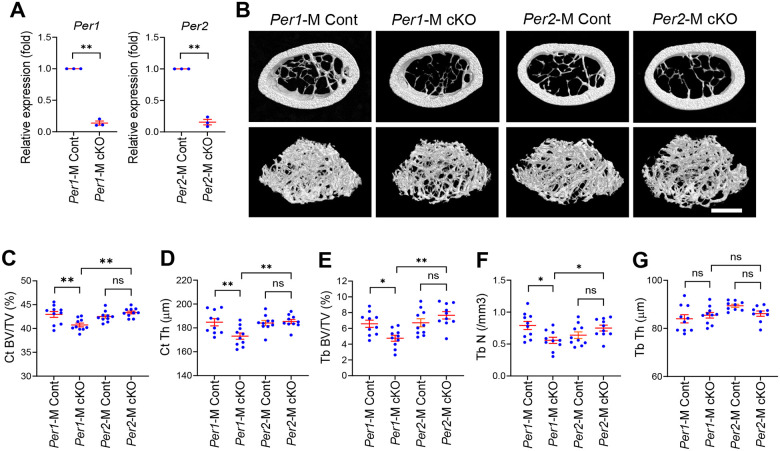
*Per1* cKO, but not *Per2* cKO, decreased bone mass in male femurs. **A.** Relative expression levels of *Per1* and *Per2* in each cKO male osteoclasts in comparison to Cont osteoclasts on day 6 quantified with qRT-PCR. n = 3. **B.** Reconstructed 3D micro-CT images of femoral midshafts (top) and distal femurs (bottom) prepared from 12-week-old male mice of the indicated genotypes. Bar, 1 mm. **C**-**G**. Quantification of the cortical bone volume/total volume ratio (**C**), cortical thickness (**D**), trabecular bone volume/total volume ratio (**E**), trabecular number (**F**), and trabecular thickness (**G**) comparing 12-week-old male mice. n = 9 or 10. Mean ± SEM is shown. ** p < 0.01, * p < 0.05, and ns for not significant with two-way ANOVA with Tukey’s method of multiple comparisons.

To investigate the roles of *Per1* and *Per2* in osteoclasts, we prepared *Cx3cr1* promoter-driven *Per1* KO mice (*Per1* cKO) alongside littermate controls (*Per1* Cont), as well as *Per2* cKO mice with controls (*Per2* Cont). qRT-PCR verified significant depletion of *Per1* (13.8 ± 3.2% remaining) and *Per2* (15.6 ± 4.4% remaining) mRNA in osteoclasts prepared from the femurs of 12-week-old male mice (Figure 1A). Micro-CT analysis of the femoral midshaft revealed a significant reduction in cortical bone volume and cortical thickness in male *Per1* cKO mice compared to those in *Per1* Cont (Figure 1B–D). Likewise, diminished distal femoral trabecular bone volume and numbers were observed in *Per1* cKO mice (Figure 1B, E, and F). In contrast, *Per2* cKO mice did not show any of these phenotypes (Figure 1B–F). Trabecular thickness was not affected in *Per1* cKO or *Per2* cKO mice (Figure 1G). In this study, we focused on male mice because none of the bone mass parameters were decreased in female *Per1* cKO and *Per2* cKO mice compared to corresponding control mice (Supplementary Figure 1A–E).

**Figure 2.**
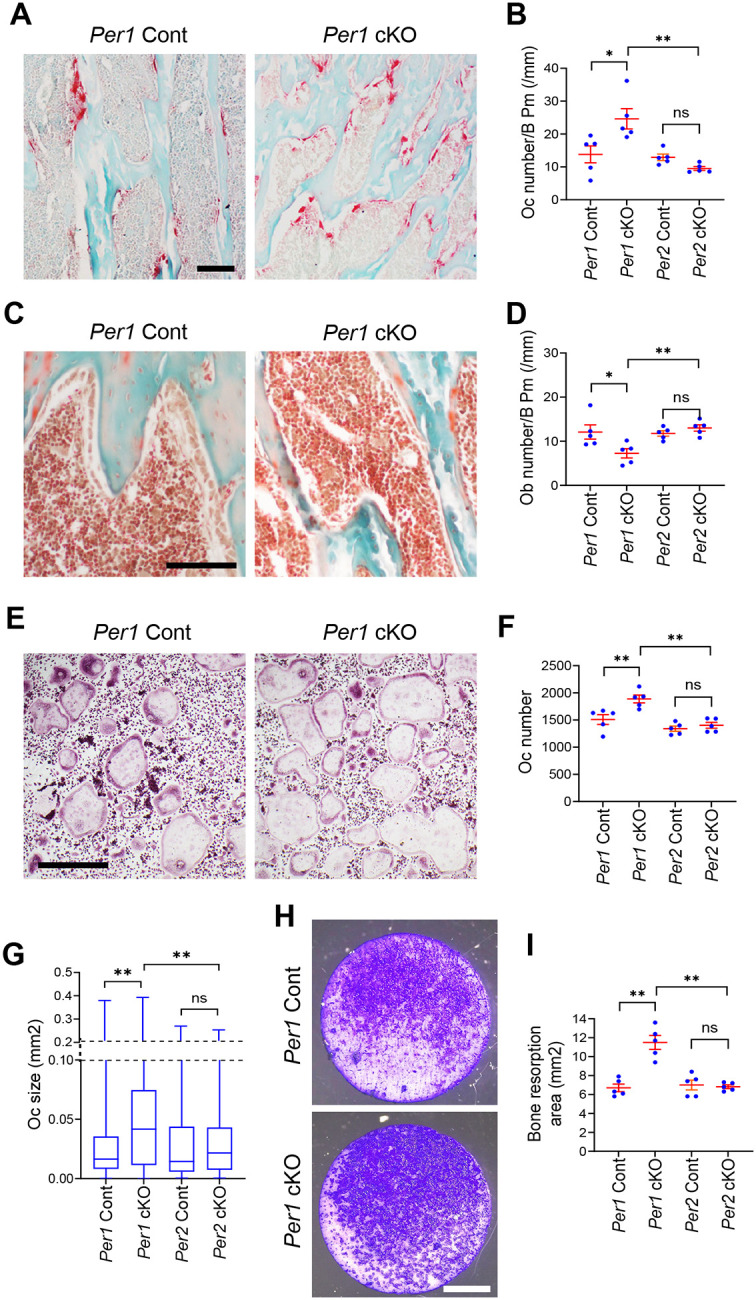
*Per1* cKO in male mice increased osteoclastogenesis but decreased osteoblastogenesis. **A.** TRAP staining of the proximal tibial sections comparing *Per1* cKO and Cont mice. Bar, 100 μm. **B.** Osteoclast numbers per bone perimeter in the proximal tibiae of the indicated genotypes. n = 5. **C.** Masson’s trichrome staining of the proximal tibial sections. Bar, 100 μm. **D.** Osteoblast numbers per bone perimeter in proximal tibiae of the indicated genotypes. n = 5. **E.** TRAP staining of osteoclasts in vitro on day 6. Bar, 500 μm. **F.** The numbers of osteoclasts per well in a 48-well plate on day 6. n = 5. **G.** Size distributions of osteoclasts on day 6. n = 3000 osteoclasts (600 osteoclasts/well x 5 wells of biologically independent experiments). **H.** Bone resorption assay stained with Toluidine blue. Bar, 2 mm. **I.** Areas of resorbed bones stained with Toluidine blue. n = 5. Histological sections and osteoclasts were prepared from 12-week-old male mice. Mean ± SEM is shown. ** p < 0.01, * p < 0.05, and ns for not significant with two-way ANOVA with Tukey’s method of multiple comparisons.

In bone histomorphometry of the proximal tibia, *Per1* cKO increased osteoclasts (Figure 2A and B) and reduced osteoblasts per bone perimeter (Figure 2C and D). The decrease in osteoblasts was likely an indirect effect because *Cx3cr1* was not substantially expressed in these cells. In contrast, the numbers of osteoclasts and osteoblasts were not changed following *Per2* cKO (Figure 2B and D, and Supplementary Figure 2A and B). The changes in the numbers of osteoclasts and osteoblasts in *Per1* cKO mice were consistent with the decreased bone mass in these mice.

### *Per1* cKO promoted osteoclastogenesis in a cell-autonomous manner

We investigated whether *Per1* depletion increased osteoclastogenesis in a cell-autonomous manner, as the *Cx3cr1* promoter was also active in monocytes and macrophages. We harvested BMMs from the femurs of 12-week-old *Per1* cKO and Cont mice and induced osteoclastogenesis with RANKL and M-CSF. Greater numbers of large-sized osteoclasts were obtained from *Per1* cKO cells compared to those from Cont cells (Figure 2E–G). Moreover, enhanced pit formation was observed on bovine bone slices caused by *Per1* cKO osteoclasts (Figure 2H and I). These phenotypes were not detected with *Per2* cKO osteoclasts (Figure 2F, G, and I, and Supplementary Figure 2C and D). These in vitro results align with the observed decrease in bone mass and increase in osteoclasts in vivo.

### Inflammatory genes were downregulated by *Per 1* cKO osteoclasts

**Figure 3.**
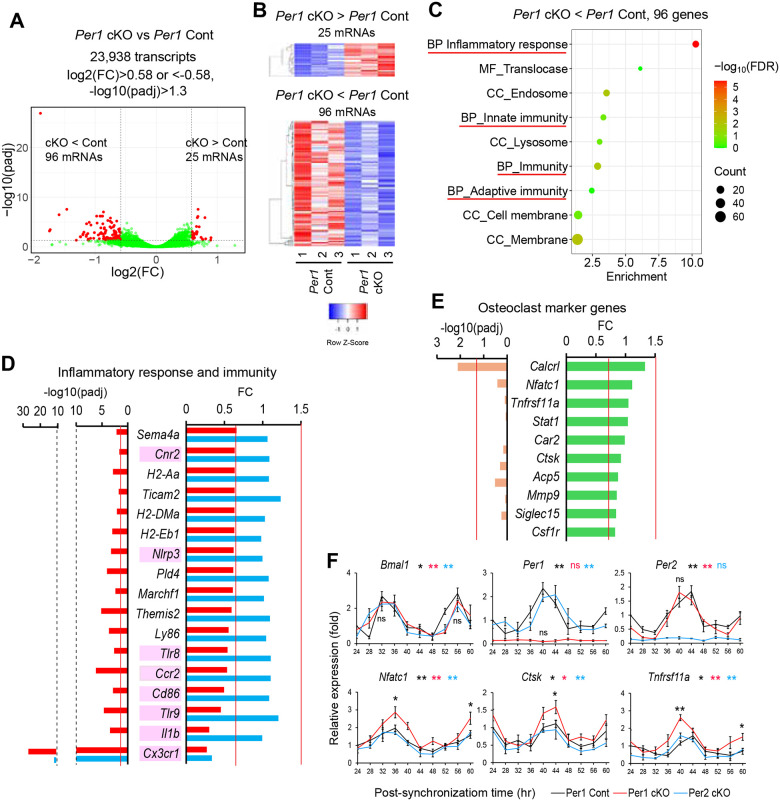
*Per1* cKO downregulated inflammatory genes in male osteoclasts. **A.** A volcano plot demonstrating differentially expressed genes between *Per1* cKO and Cont osteoclasts. FC and padj indicate fold change and adjusted p value, respectively. **B.** A heatmap displaying up- or downregulated genes in *Per1* cKO osteoclasts compared with *Per1* Cont osteoclasts. **C.** Gene ontology analysis of downregulated genes in *Per1* cKO osteoclasts compared with *Per1* Cont osteoclasts. Pathways related to inflammation and immunity are underlined in red. **D.** A list of the genes that belong to the four pathways underlined in red in (**C**). Red and blue bars indicate *Per1* cKO and *Per2* cKO osteoclasts, respectively. **E.** A list of representative osteoclasts marker genes detected with RNA-seq. **F.** Relative expression levels of circadian regulators and osteoclasts marker genes in synchronized osteoclasts comparing three genotypes. The value of *Per1* Cont cells at 24 hr was defined as 1.0 in each graph. Male osteoclasts were used in all data. (**A**) – (**E**) are based on biological triplicates, whereas (**F**) is based on biological triplicates with technical triplicated each. The red lines in (**D**) and (**E**) indicate FC = 1.5 or 0.67, and padj = 0.05. ** p < 0.01, * p < 0.05, and ns for not significant with the Cosinor analysis of circadian rhythmicity listed next to the gene names in color-coded manners in (**F**). ** p < 0.01 and * p < 0.05 with unpaired two-tailed t-test comparing peak levels of *Per1* cKO and Cont osteoclasts embedded in each graph in (**F**).

We applied RNA-seq to compare transcriptomes of *Per1* KO and Cont osteoclasts; 25 genes were upregulated and 96 were downregulated in the *Per1* cKO osteoclasts when cutoff values of > 1.5-fold difference (log2FC > 0.58 or < −0.58) and an adjusted p-value of < 0.05 (−log10(padj) > 1.3) were applied (Figure 3A and B, and Supplementary Figure 3A and B). *Per1* and *Per2* were not downregulated in each cKO cells because residual exons generated mRNA. Gene ontology (GO) analysis of the upregulated genes did not yield any enriched pathways; however, we observed enrichment of downregulated genes involved in innate and adaptive immunity, as well as inflammatory response (Figure 3C), which included 17 genes shown in Figure 3D (red bars for *Per1* cKO). RNA-seq analysis of *Per2* cKO osteoclasts identified 2 upregulated and 30 downregulated genes compared to *Per2* Cont cells (Supplementary Figure 3C and D, and 4A and B). However, there were no enriched pathways in GO analysis of these genes. In addition, the 17 genes mentioned above were not downregulated in *Per2* cKO osteoclasts (Figure 3D, blue bars), except for *Cx3cr1*, suggesting specific relevance of these genes to the increased osteoclastogenesis associated with *Per1* cKO. *Cx3cr1* was the most significantly downregulated gene because the single coding exon was replaced by the *Cre* recombinase cassette in one of the two alleles.

The 17 downregulated genes included eight genes known to affect bone mass and osteoclastogenesis (Figure 3D, highlighted in pink, and Table 1). Although germline KO was reported for some genes (*Ccr2*, *Cd86*, *Cx3cr1*, and *Tlr9*), none of them have been studied using osteoclast-specific KO mice, making it difficult to compare their mouse phenotypes with those in the current study. In vitro studies of these genes showed that they were not simple inhibitors of osteoclastogenesis, as might be expected from the *Per1* cKO phenotypes. While *Ccr2* and *Cx3cr1* promote osteoclastogenesis, other genes have dual functions (inhibition and promotion) depending on the presence or absence of inflammation (*Nlrp3*) and whether they were primed with RANKL or not (*Il1b* and *Tlr9*). The decreased bone mass and increased osteoclastogenesis observed in our study may have resulted from the cumulative effects of multiple dysregulated genes functioning at different stages of osteoclastogenesis. A case in point is the downregulation of *Cx3crl*, which was expected to inhibit osteoclastogenesis.^23^ The downregulation of other genes appeared to have counteracted this effect in *Per1* cKO and *Per2* cKO cells.

### Osteoclast marker genes were upregulated in *Per1* cKO cells

RNA-seq did not reveal the expected upregulation of osteoclast marker genes in *Per1* cKO cells despite enhanced osteoclastogenesis (Figure 3E). We tested whether this was due to the cells’ circadian rhythms not being synchronized, which masked their temporary upregulation. To test this possibility, we synchronized the circadian rhythms by incubating the cells in 50% horse serum for 1 hr and then harvested them every 4 hr over a 36-hr period. The circadian expression patterns of *Bmal1*, *Per1*, and *Per2* in *Per1* Cont cells were verified using Cosinor analysis (Figure 3F, p < 0.05 for each gene). In addition, the patterns of *Bmal1* and *Per2* expression were anti-phasic in both *Per1* cKO and Cont cells, validating successful synchronization. Moreover, *Per1* expression was substantially decreased in *Per1* cKO cells, as expected. Importantly, *Nfatc1*, *Ctsk*, and *Tnfrsf11a* exhibited circadian expression, with peak levels upregulated by *Per1* cKO, indicating that the transient upregulation of these genes in *Per1* cKO cells was obscured by the presence of cells at mixed circadian phases when the cells were not synchronized.

### *Per1* downregulated the inflammasome pathway genes

**Figure 4.**
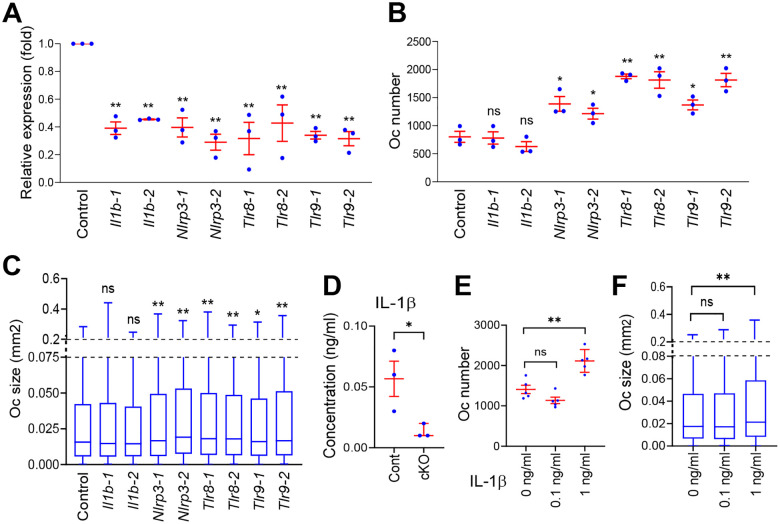
*Nlrp3*, *Tlr8*, and *Tlr9* inhibited osteoclastogenesis in vitro. **A.** Relative expression levels of the indicated genes after KD with two independent siRNA sequences each. Data are based on biological triplicates with technical triplicates each. **B.** The numbers of osteoclasts in a well of a 48-well plate after KD of the indicated genes. n = 3. **C.** Size distributions of osteoclasts on day 6. n = 3000 osteoclasts (600 osteoclasts/well x 5 wells of biologically independent experiments). **D.** Concentrations of IL-1β in the osteoclast supernatant quantified with ELISA. n = 3. **E.** The numbers of osteoclasts in a well of a 48-well plate after culture with IL-1β. n = 5. **F.** Size distributions of osteoclasts after culture with IL-1β. n = 3000 (600 osteoclasts/per well x 5 wells of biologically independent experiments). All panels except for (**D**) used male *Per1* Cont osteoclasts. Mean ± SEM is shown. ** p < 0.01, * p < 0.05, and ns for not significant with unpaired two-tailed t-test in comparison to control samples in (**A**) – (**D**) and two-way ANOVA with Tukey’s method of multiple comparisons in (**E**) and (**F**).

*Il1b*, *Tlr8*, *Tlr9*, and *Nlrp3* are involved in the inflammasome signaling pathway, which generally promotes osteoclastogenesis.^24^ However, the consequences of their depletions vary depending on the culture conditions of BMMs (Table 1). To clarify their roles in our in vitro osteoclastogenesis, we applied KD of the four genes using two independent siRNA sequences for each, which reduced each target mRNA to < 50% of the control level obtained with scrambled siRNA (Figure 4A). *Il1b* is generally pro-osteoclastogenic but it can be anti-osteoclastogenic when added into the culture prior to the addition of RANKL.^25,26^ Our KD of *Il1b* did not affect the number or size of osteoclasts (Figure 4B and C), possibly due to the low concentration of IL-1β in the supernatant of osteoclasts. Exogenous IL-1β promotes the osteoclastogenesis of BMMs at > 0.5 ng/ml in vitro.^27^ However, its concentration in the culture medium of *Per1* Cont osteoclasts was < 0.1 ng/ml, as measured by ELISA (Figure 4D); even 0.1 ng/ml was insufficient to promote osteoclastogenesis (Figures 4E and F). The low concentration was consistent with the report by Alam et al., which found no detectable IL-1β in the supernatant.^28^ Thus, IL-1β appeared to be irrelevant in our assay.

NLRP3 is a component of the inflammasome complex, which activates caspase-1, leading to the cleavage of pro-IL-1β and pro-IL-18 to produce mature IL-1β and IL-18.^24^ NLRP3 promotes osteoclastogenesis via increased IL-1β and IL-18 levels under inflammatory conditions created with LPS at 100 ng/ml; however, it inhibits osteoclastogenesis via pyroptosis under non-inflammatory conditions.^28,29^ Since LPS was undetectable (< 0.1 pg/ml) in the culture supernatants of *Per1* cKO and Cont cells on days 0 and 5, NLRP3 appears to function as an inhibitor of osteoclastogenesis in the current study. *Nlrp3* KD increased the number and size of osteoclasts, promoting osteoclastogenesis in agreement with the interpretation (Figure 4B and C). TLR9 promotes or inhibits osteoclastogenesis depending on the culture conditions, as detailed in the Discussion. In our case, the KD of *Tlr8* and *Tlr9* increased both the size and number of osteoclasts (Figure 4A–C).

### Per1-dependent circadian expression of Nlrp3, Tlr8, and Tlr9

**Figure 5.**
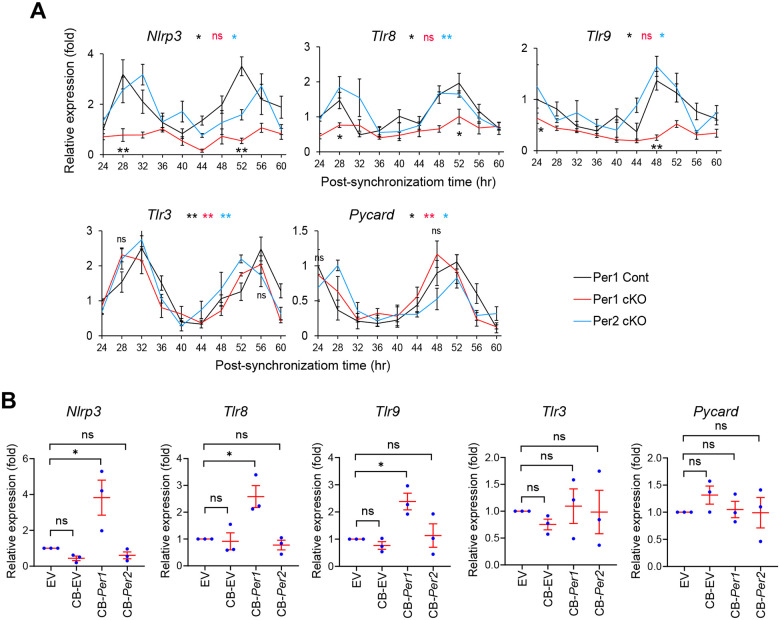
*Per1* controlled circadian expression of *Nlrp3*, *Tlr8*, and *Tlr9* in osteoclasts. **A.** Relative expression levels of the indicated genes in synchronized osteoclasts comparing *Per1* cKO, *Per2* cKO, *Per1* Cont osteoclasts. The value of *Per1* Cont cells at 24 hr was defined as 1.0 in each graph. **B.** Relative expression levels of the indicated genes after overexpression of circadian regulators in RAW264.7 cells. Abbreviations are as follows. EV: empty vector and CB: *Clock* and *Bmal1*. All experiments used male osteoclasts. Mean ± SEM is shown. ** p < 0.01, * p < 0.05, and ns for not significant with the Cosinor analysis of circadian rhythmicity listed next to the gene names in color-coded manners in (**A**). ** p < 0.01, * p < 0.05, and ns for not significant with unpaired two-tailed t-test comparing peak levels of *Per1* cKO and Cont osteoclasts in (**A**) and with two-way ANOVA with Tukey’s method of multiple comparisons in (**B**).

The circadian expressions of *Nlrp3*, *Tlr8*, and *Tlr9* were known,^30^ but whether they were dependent on *Per1* or *Per2* was unclear. qRT-PCR of synchronized cells indicated that *Per1* Cont and *Per2* cKO cells demonstrated circadian expressions of these genes (p < 0.05), while the rhythmicity was lost in *Per1* cKO cells (Figure 5A). This study included two negative control genes that were related to inflammasomes but not differentially expressed between *Per1* cKO and Cont cells: *Tlr3* (recognizes viral double-stranded RNA, log2(FC) = −0.3, and −log10(padj) = 0.17) and *Pycard* (a component of inflammasomes, log2(FC) = −0.06, and −log10(padj) = 0.05).^31^ These genes also exhibited circadian expression, which was not disrupted by *Per1* KO, underscoring the specific regulation of *Nlrp3*, *Tlr8*, and *Tlr9* by *Per1*. In a complementary experiment, we transduced *Clock*, *Bmal1*, *Per1*, and *Per2* in various combinations into the macrophage cell line RAW264.7 because plasmid transfection with osteoclasts was inefficient. The transfection of *Clock* and *Bmal1* did not increase the expression levels of *Nlrp3*, *Tlr8*, or *Tlr9* compared to that with the empty vector when the same total amount of plasmids was used (Figure 5B). However, these genes were upregulated when *Per1*, but not *Per2*, was included. The expression levels of the two control genes (*Tlr3* and *Pycard*) were not affected by the transfections. Collectively, these results supported our interpretation of the *Per1*-specific upregulation of *Nlrp3*, *Tlr8*, or *Tlr9*.

## Discussion

This study demonstrated that *Per1* was a cell-autonomous inhibitor of osteoclastogenesis in vitro, likely achieved by the upregulation of a group of inflammatory genes known to control osteoclastogenesis in context-dependent manners. These roles could explain the decreased bone mass in *Per1* cKO mice, suggesting the presence of the *Per1*–inflammatory genes–osteoclast axis. This axis appears to be influenced by a sex-based bias since bone mass was not decreased in female *Per1* cKO mice, which requires further studies. In contrast to *Per1*, cKO of *Per2* did not affect bone mass or osteoclastogenesis despite sharing a high similarity at the amino acid level (73.4%) with *Per1*. Our finding is the second example of *Per1*-specific regulation of inflammatory genes, following an example that we reported in macrophages, although the target inflammatory genes were different.^12^ The relationship between circadian rhythm disruption and bone loss has been empirically established; however, direct connections between specific circadian regulators and osteoclasts remain unknown in vivo, except for the controversial roles of *Bmal1* mentioned earlier. In addition, the involvement of inflammatory genes was not discussed in the studies on *Bmal1* KO.

We selected the *Cx3cr1* promoter to drive the *Cre* gene, instead of the *Ctsk* promoter because *Ctsk* is also expressed in periosteal mesenchymal stem cells, which can differentiate into osteoblasts,^32^ thereby compounding the interpretation of our results. However, the *Cx3cr1* is also expressed in the cells other than the monocyte/macrophage lineages, such as dendritic cells, natural killer (NK) cells, and some T cell subtypes.^33^ Depletion of *Per1* in these cells is also known to affect bone remodeling via secreted cytokines. For example, *Per1* KO in NK cells alters the peak level and timing of the circadian expression of interferon γ (IFN-γ) and cytolytic factors (perforin and granzyme B).^34^ *Per1* KD in helper T cells inhibits the synthesis of IFN-γ, IL-2, and TNF-α.^35^ These cytokines are involved in bone remodeling and osteoporosis.^36–38^ Furthermore, *Per1* depletion indirectly affected *Cx3cr1*-negative cells as indicated by the decreased number of osteoblasts in the tibiae. Recent advances in single-cell spatial transcriptomics may enable us to analyze the contributions of each cell type. This approach would also enable us to verify whether the downregulated genes in the RNA-seq were reproducible in vivo, which is particularly significant for the dual-function genes *Nlpr3*, *Tlr9*, and potentially *Tlr8*.

TLR8 and TLR9 belong to the pattern recognition receptors in the innate immune system, which are localized on the membranes of endosomes, lysosomes, and endolysosomes.^31^ TLR8 binds to single-stranded RNA, including viral RNA, whereas TLR9 binds to unmethylated CpG-containing DNA, which is more abundant in viral and bacterial DNA than in mammalian DNA. Binding to these molecules leads to the production of pro-inflammatory cytokines (such as IL-1β and TNF-α) and type I interferons (such as IFN-α and IFN-β), which are generally pro-osteoclastogenic. TLR9 inhibits osteoclastogenesis when it is activated in the presence of M-CSF and RANKL at the start of culture, but promotes osteoclastogenesis when activated after the cells have been primed by M-CSF and RANKL for three days.^39,40^ The inhibition of osteoclastogenesis by TLR9 has been explained by the enhanced degradation of the c-fos protein, an essential inducer of osteoclastogenesis.^41^ However, the relevance of this culture condition-dependent difference in osteoclastogenesis to physiological osteoclastogenesis in vivo remains unclear. In the present study, TLR9 activation was not induced, although the possibility of unintentional activation could not be excluded. RANKL was added to the culture medium from day 2, and *Tlr9* siRNA was introduced on day 4, leading to enhanced osteoclastogenesis. This promotion likely reflects the role of TLR9 at the baseline (non-activated) level, which could change once it is activated under an inflammatory condition. Although TLR8 has not been characterized in detail, it likely has similar roles due to the shared downstream signaling pathways.

The involvement of several genes relevant to inflammasomes (*Tlr8*, *Tlr9*, *Nlrp3*, and *Il1b*) in osteoclastogenesis raises the possibility of a crosstalk between osteoclasts and immune cells (osteoimmunology)^42^ as a part of the *Per1*-mediated circadian regulation of bone remodeling. The consequence of crosstalk would become amplified when inflammasomes are activated by microorganisms or cell debris. This may impact inflammatory bone diseases influenced by circadian rhythms, such as rheumatoid arthritis and osteoarthritis, where osteoclast-mediated bone destruction is a significant factor.^6,43^ Further mechanistic studies on *Per1*-specific circadian regulation of osteoimmunology could lead to the identification of novel therapeutic options for the inflammatory bone diseases, in addition to non-inflammatory predispositions to osteoporosis caused by circadian disruption, as broader implications of this research.

## Supplementary Material

Supplement 1

Supplement 2

## Figures and Tables

**Table 1.** The roles of selected genes in osteoclastogenesis

Gene	Relevance to osteoclastogenesis	Ref
*Ccr2*	C-C motif chemokine receptor 2. Increased bone mass and decreased osteoclasts in *Ccr2*^*−/−*^ mice. Inhibited osteoclastogenesis from *Ccr2*^*−/−*^ cells in vitro.	^44,45^
*Cd86*	Decreased bone mass and increased osteoclasts in *Cd80*^−/−^;*Cd86*^−/−^ mice. Normal osteoclastogenesis by *Cd80*^−/−^;*Cd86*^−/−^ monocytes in vitro. CD80 and CD86 are closely related.	^46^
*Cnr2* (*CB2*)	Cannabinoid receptor 2. Uncertain roles. A study showed decreased bone mass and increased osteoclasts in *CB* ^*−/−*^ mice, while another indicated normal bone mass.	^47,48^
*Cx3cr1*	Increased bone mass in *Cx3cr1*^*−/−*^ mice and impaired osteoclastogenesis in vitro.	^23^
*Il1b*	IL-1β. Generally pro-osteoclastogenesis but can be anti-osteoclastogenesis depending on the culture conditions in vitro.	^25,26^
*Nlrp3*	NOD-, LRR- and pyrin domain-containing protein 3a. Inhibits osteoclastogenesis under physiological conditions in vitro but promotes it during infection.	^28,29^
*Tlr8*	Toll-like receptor 8. Binds to bacterial and viral single-stranded RNA. Promotes osteoclastogenesis when stimulated by a ligand in vitro. Stage-specific effect on osteoclastogenesis (below) is not known.	^49^
*Tlr9*	Recognizes DNA of bacteria and viruses and activates inflammation. Increased osteoclastogenesis and bone resorption in *Tlr9*^*−/−*^ mice as a secondary effect of systemic inflammation. Inhibits or promotes osteoclastogenesis depending on the stage of differentiation.	^39,40,50^

## Data Availability

RNA-seq data have been deposited to Gene Expression Omnibus (GEO) under the accession number of GSE283894 (*Per1* cKO and control) and GSE292534 (*Per2* cKO and control).
