# Supplementary material for: The circadian regulator PER1 inhibits osteoclastogenesis by activating inflammatory genes
Source: bioRxiv. 2025 Sep 19:2025.09.18.677145. Preprint. [Version 1] doi: 10.1101/2025.09.18.677145 (PMC12458312; doi:10.1101/2025.09.18.677145)
Supplement: Supplement 1 [file media-1.pdf]

Graphical abstract

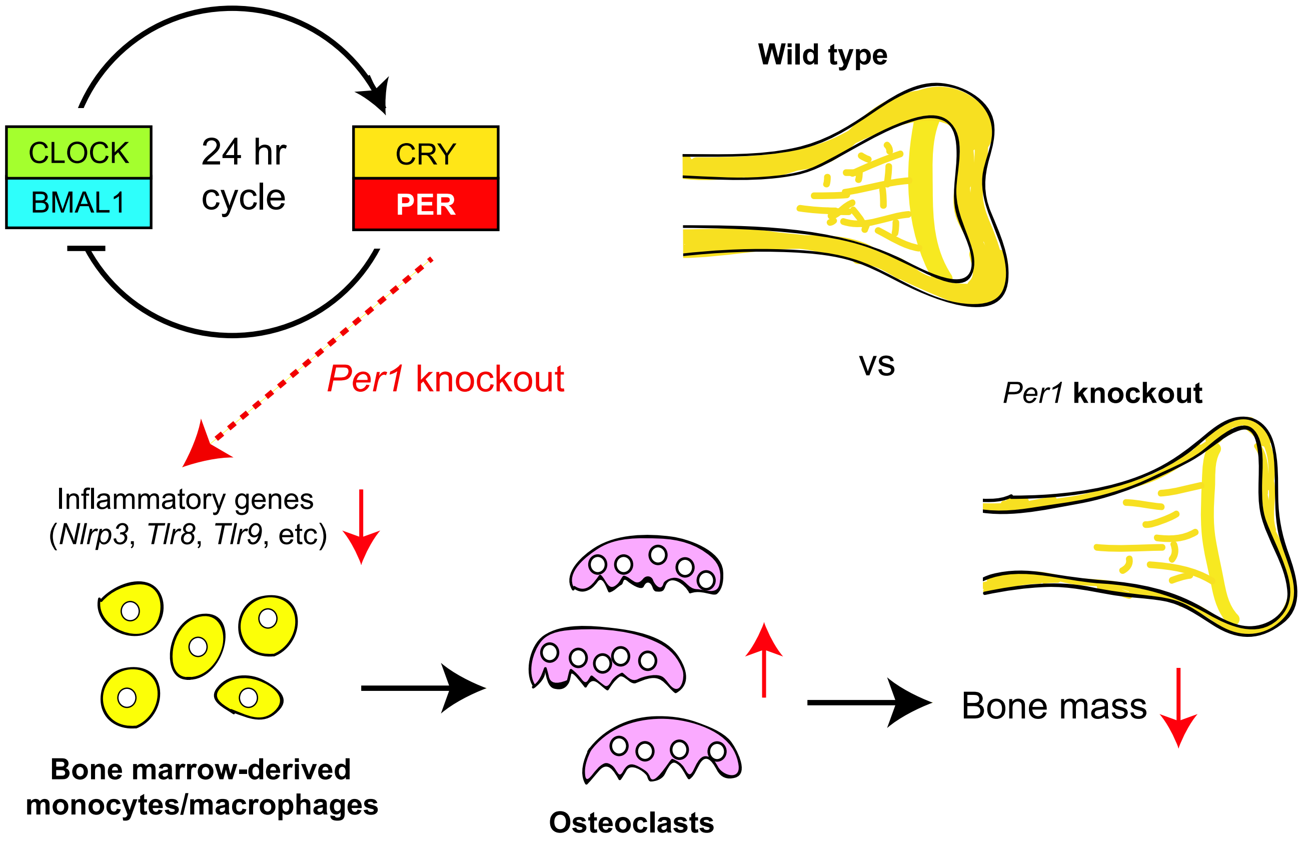

**Graphical abstract**

The mammalian CLOCK/BMAL1 heterodimer activates hundreds of target genes including *Cry* and *Per*, whose proteins inhibit CLOCK/BMAL1, thus generating circadian rhythms with a 24-hr period. When *Per1* is knocked out, inflammatory genes are downregulated in bone marrow-derived monocytes and macrophages, which promotes osteoclastogenesis and decreases bone mass.
