# Supplementary material for: The circadian regulator PER1 inhibits osteoclastogenesis by activating inflammatory genes
Source: bioRxiv. 2025 Sep 19:2025.09.18.677145. Preprint. [Version 1] doi: 10.1101/2025.09.18.677145 (PMC12458312; doi:10.1101/2025.09.18.677145)
Supplement: Supplement 2 [file media-2.pdf]

Supplementary Material

The circadian regulator PER1 inhibits osteoclastogenesis by activating inflammatory genes

Nobuko Katoku-Kikyo[1,2], Elizabeth K. Vu[1,3], Samuel Mitchell[1,3], Ismael Y. Karkache[1,3], Elizabeth W. Bradley[1,3,*], and Nobuaki Kikyo[1,2,*]

[1] Stem Cell Institute, University of Minnesota

[2] Department of Genetics, Cell Biology, and Development, University of Minnesota

[3]Department of Orthopedic Surgery, University of Minnesota

[*] Corresponding authors

Email: ebradle1@umn.edu (EWB) and kikyo001@umn.edu (NK).

List of Supplementary Material

Supplementary Figure 1-4

Supplementary Figure Legends

Supplementary Table 1-3

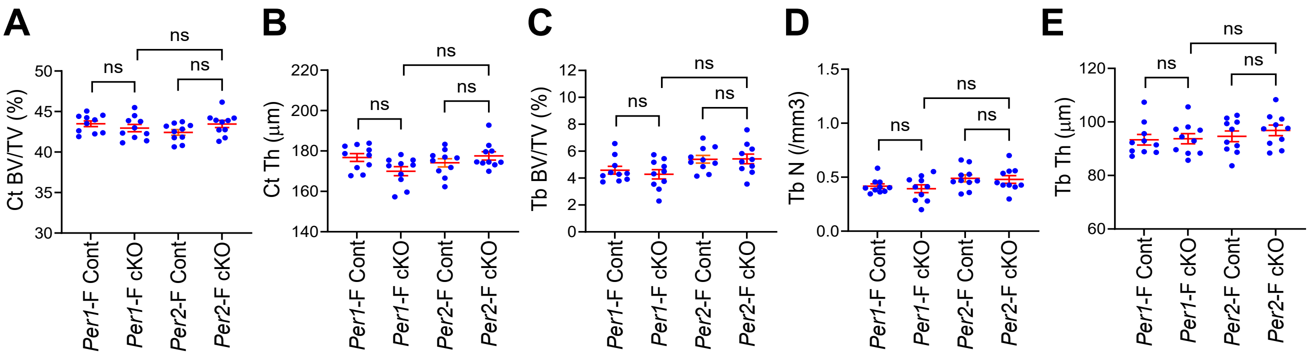
A
B
C
D
E
Ct BV/TV (%)
Ct Th (μm)
Tb BV/TV (%)
Tb N (/mm3)
Tb Th (μm)
ns
Per1-F Cont
Per1-F cKO
Per2-F Cont
Per2-F cKO

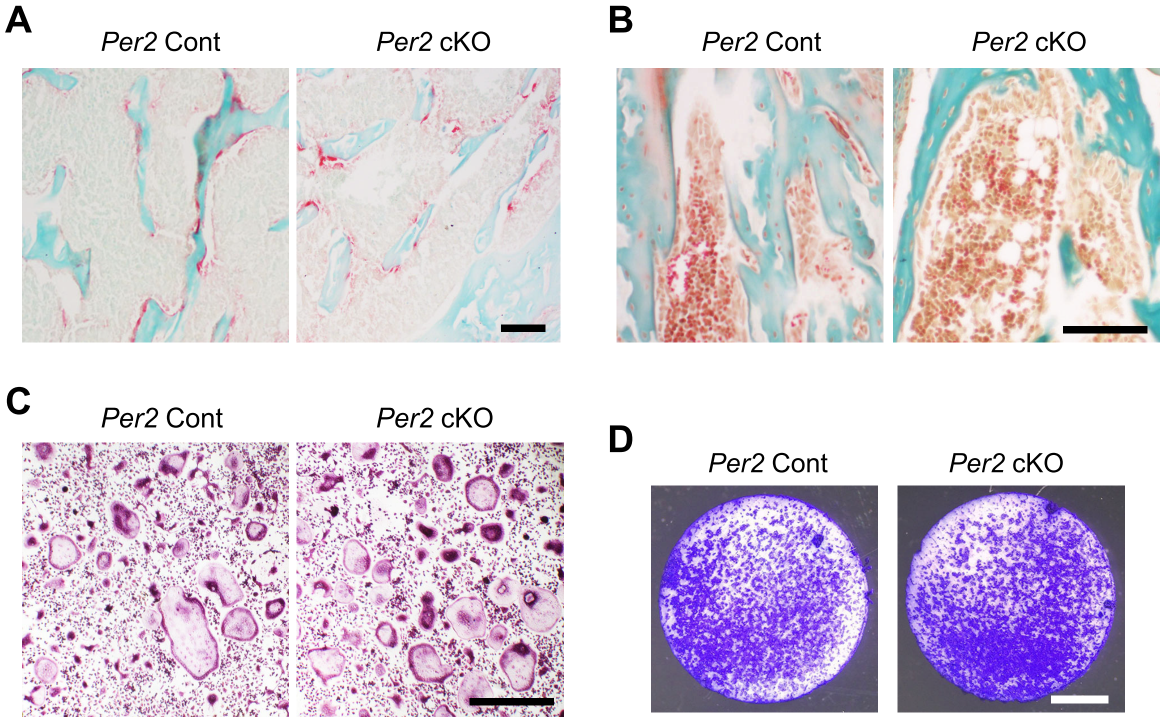
A
Per2 Cont
Per2 cKO
B
Per2 Cont
Per2 cKO
C
Per2 Cont
Per2 cKO
D
Per2 Cont
Per2 cKO

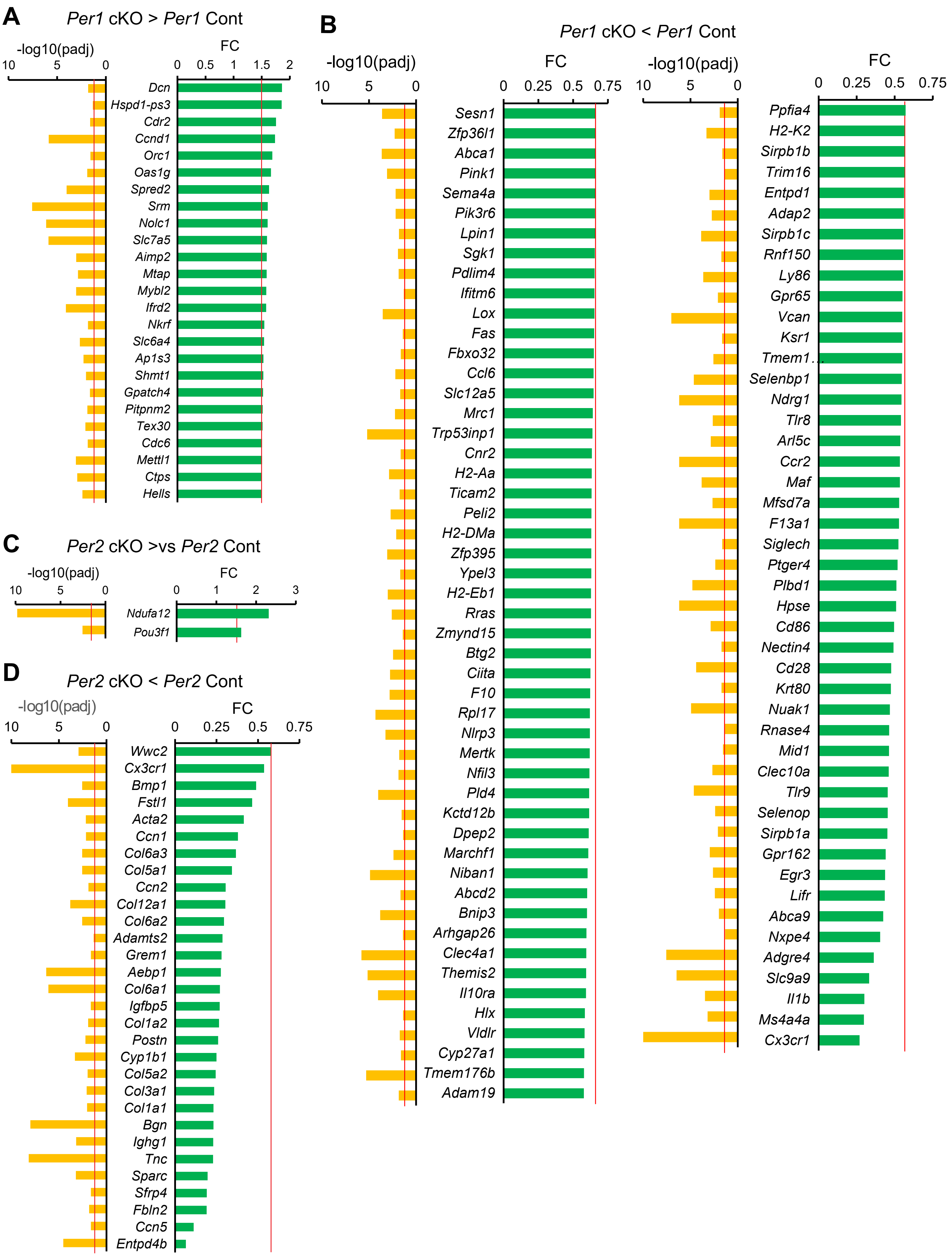
A
Per1 cKO > Per1 Cont
-log10(padj)
FC
Dcn
Hspd1-ps3
Cdr2
Ccnd1
Orc1
Oas1g
Spred2
Srm
Nolc1
Slc7a5
Aimp2
Mtap
Mybl2
Ifrd2
Nkrf
Slc6a4
Ap1s3
Shmt1
Gpatch4
Pitpnm2
Tex30
Cdc6
Mettl1
Ctps
Hells
B
Per1 cKO < Per1 Cont
Sesn1
Zfp36l1
Abca1
Pink1
Sema4a
Pik3r6
Lpin1
Sgk1
Pdlim4
Ifitm6
Lox
Fas
Fbxo32
Ccl6
Slc12a5
Mrc1
Trp53inp1
Cnr2
H2-Aa
Ticam2
Peli2
H2-DMa
Zfp395
Ypel3
H2-Eb1
Rras
Zmynd15
Btg2
Ciita
F10
Rpl17
Nlrp3
Mertk
Nfil3
Pld4
Kctd12b
Dpep2
Marchf1
Niban1
Abcd2
Bnip3
Arhgap26
Clec4a1
Themis2
Il10ra
Hlx
Vldlr
Cyp27a1
Tmem176b
Adam19
Ppfia4
H2-K2
Sirpb1b
Trim16
Entpd1
Adap2
Sirpb1c
Rnf150
Ly86
Gpr65
Vcan
Ksr1
Tmem1...
Selenbp1
Ndrg1
Tlr8
Arl5c
Ccr2
Maf
Mfsd7a
F13a1
Siglech
Ptger4
Plbd1
Hpse
Cd86
Nectin4
Cd28
Krt80
Nuak1
Rnase4
Mid1
Clec10a
Tlr9
Selenop
Sirpb1a
Gpr162
Egr3
Lifr
Abca9
Nxpe4
Adgre4
Slc9a9
Il1b
Ms4a4a
Cx3cr1
C
Per2 cKO >vs Per2 Cont
Ndufa12
Pou3f1
D
Per2 cKO < Per2 Cont
Wwc2
Cx3cr1
Bmp1
Fstl1
Acta2
Ccn1
Col6a3
Col5a1
Ccn2
Col12a1
Col6a2
Adamts2
Grem1
Aebp1
Col6a1
Igfbp5
Col1a2
Postn
Cyp1b1
Col5a2
Col3a1
Col1a1
Bgn
Ighg1
Tnc
Sparc
Sfrp4
Fbln2
Ccn5
Entpd4b

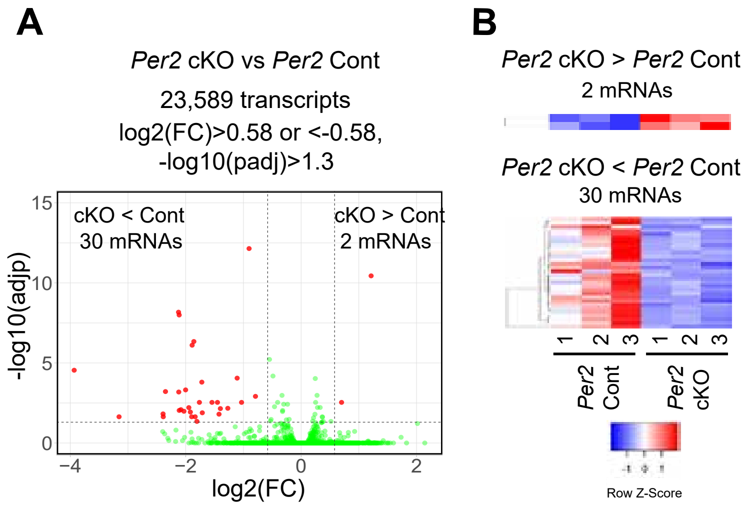
A
Per2 cKO vs Per2 Cont
23,589 transcripts
log2(FC)>0.58 or <-0.58,
-log10(padj)>1.3
cKO < Cont
30 mRNAs
cKO > Cont
2 mRNAs
-log10(adjp)
log2(FC)
B
Per2 cKO > Per2 Cont
2 mRNAs
Per2 cKO < Per2 Cont
30 mRNAs
Per2 Cont
Per2 cKO
Row Z-Score

Supplementary Figure Legends

Supplementary Figure 1. Bone mass of the female femurs was not affected by *Per1* or *Per2* cKO.

A-**E**. Quantification of the cortical bone volume/total volume ratio (**A**), cortical thickness (**B**), trabecular bone volume/ total volume ratio (**C**), trabecular number (**D**), and trabecular thickness (**E**) comparing 12-week-old female mice. n = 9 or 10.

ns for not significant with two-way ANOVA with Tukey's method of multiple comparisons.

Supplementary Figure 2. *Per2* cKO in male mice did not affect osteoclastogenesis or osteoblastogenesis.

A. TRAP staining of the proximal tibial sections comparing *Per2* cKO and Cont mice. Bar, 100 μm.

B. Masson's trichrome staining of the proximal tibial sections. Bar, 100 μm.

C. TRAP staining of osteoclasts on day 6. Bar, 500 μm.

D. Bone resorption assay stained with Toluidine blue. Bar, 2 mm.

Histological sections and osteoclasts were prepared from 12-week-old male mice.

Supplementary Figure 3. Lists of genes differentially expressed between cKO and control osteoclasts of *Per1* and *Per2* each.

A and **B.** Genes up- (**A**) and downregulated (**B**) in *Per1* cKO osteoclasts compared with *Per1* Cont osteoclasts.

C and **D.** Genes up- (**C**) and downregulated (**D**) in *Per2* cKO osteoclasts compared with *Per2* Cont osteoclasts.

All data are based on n = 3 of male cells. The red lines indicate FC = 1.5 or 0.67, and padj = 0.05.

Supplementary Figure 4. *Per2* cKO dysregulated smaller numbers of genes compared with *Per1* cKO.

A. A volcano plot demonstrating differentially expressed genes between *Per2* cKO and Cont osteoclasts.

B. A heatmap displaying up- or downregulated genes in *Per2* cKO osteoclasts compared with *Per2* Cont osteoclasts.

All data are based on n = 3 male osteoclasts.

Supplementary Table 1. Sequences of genotyping primers and the sizes of the PCR products

Gene	Sequence
Cx3cr1 forward for the wild type allele	CCTCAGTGTGACGGAGACAG
Cx3cr1 forward for the *Cre* allele	GACATTTGCCTTGCTGGAC
Cx3cr1 reverse	GCAGGGAAATCTGATGCAAG
Sizes of PCR products of *Cx3cr1*	302 bp for the wild type allele and 380 bp for the *Cre* allele
Per1 forward	ATGAAGGTGGATAGGCTAGGGC
Per1 reverse	GCCTTACCTTTCATCTACATCCTGG
Sizes of PCR products of *Per1*	459 bp for the wild type allele and 539 bp for the floxed allele
Per2 forward	GGGACCTGACCCATCATTCT
Per2 reverse for the wild type allele	TAGGCTTCACCACAGGGTTC
Per2 reverse for the floxed allele	GAACTTCGGAATAGGAACTTCG
Sizes of PCR products of *Per2*	248 bp for the wild type allele and 147 bp for the floxed allele

Supplementary Table 2. Sequences of qPCR primers

Gene	Forward	Reverse
Gapdh	TGCACCACCAACTGCTTAG	GATGCAGGGATGATGTTC
Bmal1	CAACCCATACACAGAAGCAAAC	CATCTGCTGCCCTGAGAATTA
Per1	CCTGGAGGAATTGGAGCATATC	CCTGCCTGCTCCGAAATATAG
Per2	CAAAGCTGACGCACACAAAG	TTAGCCTTCACCTGCTTCAC
Il1b	CCACCTCAATGGACAGAATATCA	CCCAAGGCCACAGGTATTT
Nlrp3	GTTCTGAGCTCCAACCATTCT	CACTGTGGGTCCTTCATCTTT
Tlr8	GTAACGCACCGTCTAGGATTT	TTCAGCTCACTTTCCTCTGTG
Tlr9	TGGACGGGAACTGCTACTA	CAGAGACAGATGGGTGAGATTG
Tlr3	ACCTCCAGAAGAACCTCATAAC	GAACGGATTGAAGCGCATATC
Pycard	ACCAGCCAAGACAAGATGAG	CCATCACCAAGTAGGGATGTATT

Supplementary Table 3. Target DNA sequences of siRNAs

Gene	Target DNA sequence
Non-targeting control	TGGTTTACATGTCGACTAA
Il1b-1	GAGGACATGAGCACCTTCTTT
Il1b-2	GCAGGCAGTATCACTCATTGT
Nlrp3-1	GCCATGTGGAGATCCTAGGTT
Nlrp3-2	GGAGTTCTTCGCTGCTATGTA
Tlr8-1	GAACCAGTGTTACAGTACTCA
Tlr8-2	GGATTTAACCAACAACAGACT
Tlr9-1	GGACAGGTGTAAGAACTTCAA
Tlr9-2	GTTCAGTGAGCTACCACAGTT
